# Mesenchymal stem cells: current clinical progress in ARDS and COVID-19

**DOI:** 10.1186/s13287-020-01804-6

**Published:** 2020-07-22

**Authors:** Kun Xiao, Fei Hou, Xiuyu Huang, Binbin Li, Zhi Rong Qian, Lixin Xie

**Affiliations:** 1 Department of Pulmonary and Critical Care Medicine, Chinese People’s Liberation Army (PLA) General Hospital, Beijing, China; 2 Medical School of Chinese People’s Liberation Army (PLA), Beijing, China; 3 Scientific Research Center, The Seventh Affiliated Hospital, Sun Yat-sen University, Shenzhen, Guangdong China

**Keywords:** Acute respiratory distress syndrome, Cell therapy, Mesenchymal stem cells, COVID-19, Clinical trials

## Abstract

Acute respiratory distress syndrome (ARDS) develops rapidly and has a high mortality rate. Survivors usually have low quality of life. Current clinical management strategies are respiratory support and restricted fluid input, and there is no suggested pharmacological treatment. Mesenchymal stromal cells (MSCs) have been reported to be promising treatments for lung diseases. MSCs have been shown to have a number of protective effects in some animal models of ARDS by releasing soluble, biologically active factors. In this review, we will focus on clinical progress in the use of MSCs as a cell therapy for ARDS, which may have clinical implications during the coronavirus disease 2019 (COVID-19) pandemic.

## Introduction

### SARS-CoV-2-induced ARDS

**Fig. 1:**
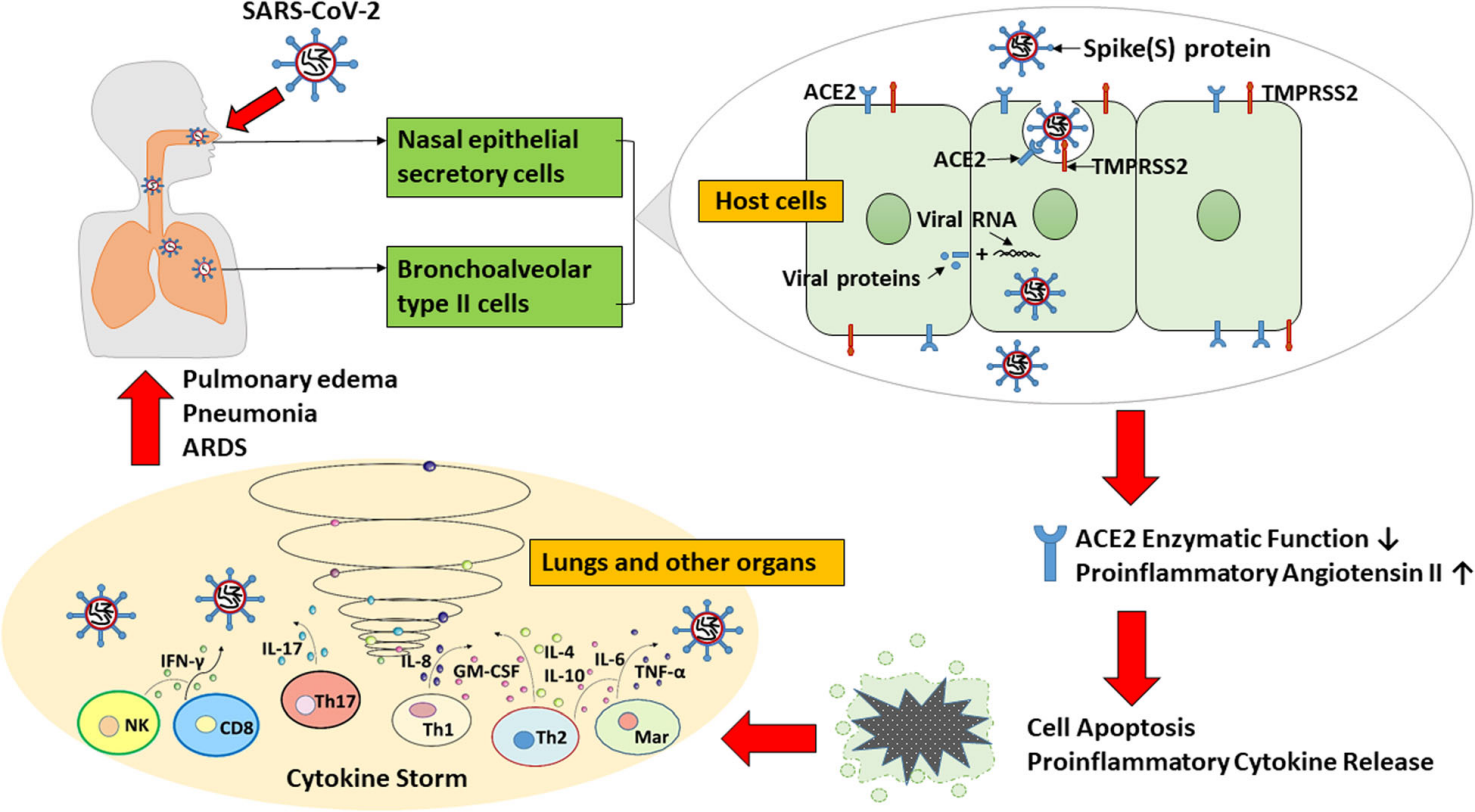
Schematic diagram of how SARS-CoV-2 causes COVID-19. The SARS-CoV-2 virus enters the respiratory tract. The S protein on the surface of the virus binds to the secretory cells of the nasal epithelium and the membrane protein ACE2, which is highly expressed in bronchoalveolar type II cells. Subsequently, SARS-CoV-2 enters the host cell through phagocytosis, thereby partially reducing or completely abrogating the enzymatic function of ACE2 and increasing the concentration of proinflammatory angiotensin II. A high concentration of angiotensin II in the lung interstitium promotes apoptosis, releases proinflammatory cytokines, and triggers an inflammatory response [9], leading to symptoms of a cytokine storm and pneumonia in COVID-19 patients and to ARDS in severe cases. Mar, macrophage; ACE2, angiotensin-converting enzyme 2; TMPRSS2, transmembrane protease serine 2; NK, natural killer cell; IFN, interferon; IL, interleukin; GM-CSF, granulocyte-macrophage colony-stimulating factor; TNF, tumor necrosis factor

Acute respiratory distress syndrome (ARDS) refers to the diffuse damage to the pulmonary capillary endothelium and alveolar epithelium caused by infection, mechanical stimulation, shock, blood transfusion, and other factors and is the main cause of poor prognosis in critically ill patients [1]. The pathological changes associated with ARDS are mainly alveolar-capillary membrane damage, increased permeability leading to inflammatory exudate in the alveoli, epithelial and interstitial edema, pulmonary edema, carbon dioxide diffusion disorders, and gas exchange disorders. This pathology leads to intractable hypoxemia, which eventually leads to respiratory distress [2]. A recent worldwide outbreak of pneumonia caused by severe acute respiratory syndrome coronavirus 2 (SARS-CoV-2) ranges from asymptomatic or mild upper respiratory tract infection to severe pneumonia, ARDS, or even death. The possible mechanism is shown in Fig. 1. The nasal epithelium was found to be the first site of infection and transmission among individuals with symptomatic and asymptomatic SARS-CoV-2 infections [3]. Similar to SARS-CoV, the S protein of SARS-CoV-2 binds to angiotensin-converting enzyme 2 (ACE2) and enters cells in a manner catalyzed by transmembrane protease serine 2 (TMPRSS2) protease in type II alveolar epithelial cells [4, 5]. The affinity of the S protein of SARS-CoV-2 for ACE2 is the main determinant of the replication rate of SARS-CoV-2 and the severity of COVID-19 disease [6]. Despite significant advances in supportive treatment techniques such as mechanical ventilation, the incidence and mortality of ARDS remain high. An observational study in Jinyintan Hospital in Wuhan, China, found that a majority (67–85%) of critically ill patients with SARS-CoV-2 infections developed ARDS and that the mortality was as high as 61.5% in patients with ARDS [7, 8]. Thus, effective control and treatment of ARDS are major challenges that all medical units are facing to suppress the mortality ratio of COVID-19 patients. However, to the best of our knowledge, there are still no approved medicines for ARDS; thus, the development of effective treatment strategies or agents is highly desired. Among these, the administration of MSCs is considered to be a promising approach for the treatment of ARDS in different respiratory viral infection models. This review discusses in detail the current laboratory and clinical research progress on the use of MSCs in the treatment of ARDS caused by SARS-CoV-2 and other respiratory infectious viruses and aims to provide a comprehensive view to facilitate future development of treatments in the battle against COVID-19.

## MSCs

MSCs are considered to have broad clinical application prospects, including as a treatment for ARDS. MSCs have broad bioactivities, including repair, immunomodulation, increased alveolar fluid clearance, and regulation of pulmonary vascular endothelial permeability [10]. Although MSCs have a wide range of sources, such as the bone marrow, umbilical cord, fat, amniotic membrane, menstrual blood, and other tissues [11], MSCs from different sources have significant similarities, such as being adherent and teardrop- or spindle-shaped [12]. MSCs have the advantages of self-renewal, multidirectional differentiation, and immunosuppression. These cells differentiate into osteocytes, chondrocytes, and adipocytes in the appropriate induction medium [13]. Various soluble factors secreted by MSCs have been shown to be related to immune regulation [14]. Extracellular vesicles, including exosomes, have shown strong abilities to repair, regenerate, and protect against various organ injuries [15, 16], which may play important roles in the treatment of ARDS. In addition, due to the low homing rate of MSCs after infusion, the migration of MSCs to the damaged site is also the focus of current research [17]. As a possible mechanism, CD90 binding to the specific integrins b3 and b5 can promote MSC homing. Activation of integrin b1 or b5 has previously been shown in lung injury models [18, 19]. Studies have reported using transgenic MSCs to improve the homing effect [20], pretreating MSCs with hypoxia [21], activating the FAK/ERK pathway [22], and using nanotechnology to build carriers [23], and so on.

## MSCs in COVID-19

The team from Wuhan Central Hospital analyzed the data of 109 patients diagnosed with ARDS and showed that ARDS progressed faster than other respiratory illnesses and that treatment was difficult. In general, the mortality rate of COVID-19 patients with ARDS was high. When the ARDS level became moderate and severe, the mortality rate climbed as high as 70% [24]. Because stem cells are resistant to tissue damage, promote tissue repair, and have immunoregulatory effects, research by scientists worldwide in the field of stem cells is bringing hope for a treatment for respiratory virus-induced pneumonia [25]. In the H9N2-infected mouse model, MSC treatment increased the survival rate and decreased lung edema and histological injury compared to those of the placebo group. MSCs improved gas exchange and reduced the levels of BALF chemokines and cytokines, including GM-CSF, MIG, IL-1α, IFN-γ, IL-6, and TNF-α [26]. Additionally, MSCs were also reported to be effective in treating an H1N1-infected pig model, and the MSC-treated group had decreased viral shedding in nasal swabs and reduced viral replication in the lungs. The virus-induced production of proinflammatory cytokines, including TNF-α and CXCL-10, was inhibited after MSC treatment [27]. Considering the positive results in various respiratory virus-induced pneumonia cases, MSCs will probably also by effective against SARS-CoV-2 virus, especially by reducing the risk of cytokine storms, which cause ARDS and organ failure in patients with severe disease.

At present, over 20 clinical trials of MSCs in treating COVID-19 are in progress, including clinical trials based on MSC derivatives. In a clinical study involving seven COVID-19 pneumonia patients, including elderly patients, in Beijing Youan Hospital, China, MSC transplantation apparently improved the outcomes of all patients by 14 days after MSC injection without adverse effects. Patient pulmonary function and symptoms improved only 2 days after MSC injection. Cytokine-secreting immune cells, including CXCR3+CD4+ T cells, CXCR3+CD8+ T cells, and CXCR3+ NK cells, disappeared within 1 week. The level of the proinflammatory cytokine TNF-α was significantly decreased, which indicated the great potential of MSCs in treating patients with severe ARDS [28].

In China, on January 27, the First Affiliated Hospital of Zhejiang University Medical College announced the use of stem cells to treat patients with severe disease. In addition, there are currently 9 registered clinical studies of mesenchymal stem cells for the treatment of acute lung injury (ALI) or ARDS. The advantages and potential of mesenchymal stem cells in defending against severe COVID-19 pneumonia-induced ALI or ARDS have been confirmed [29]. Another study found that after infection with COVID-19, MSC treatment inhibited the overactivation of the immune system and promoted endogenous repair by improving the lung microenvironment. However, studies in a larger patient population are needed to further validate MSC therapeutic interventions [30].

## Mechanisms of the therapeutic benefits of MSCs in ARDS

**Fig. 2:**
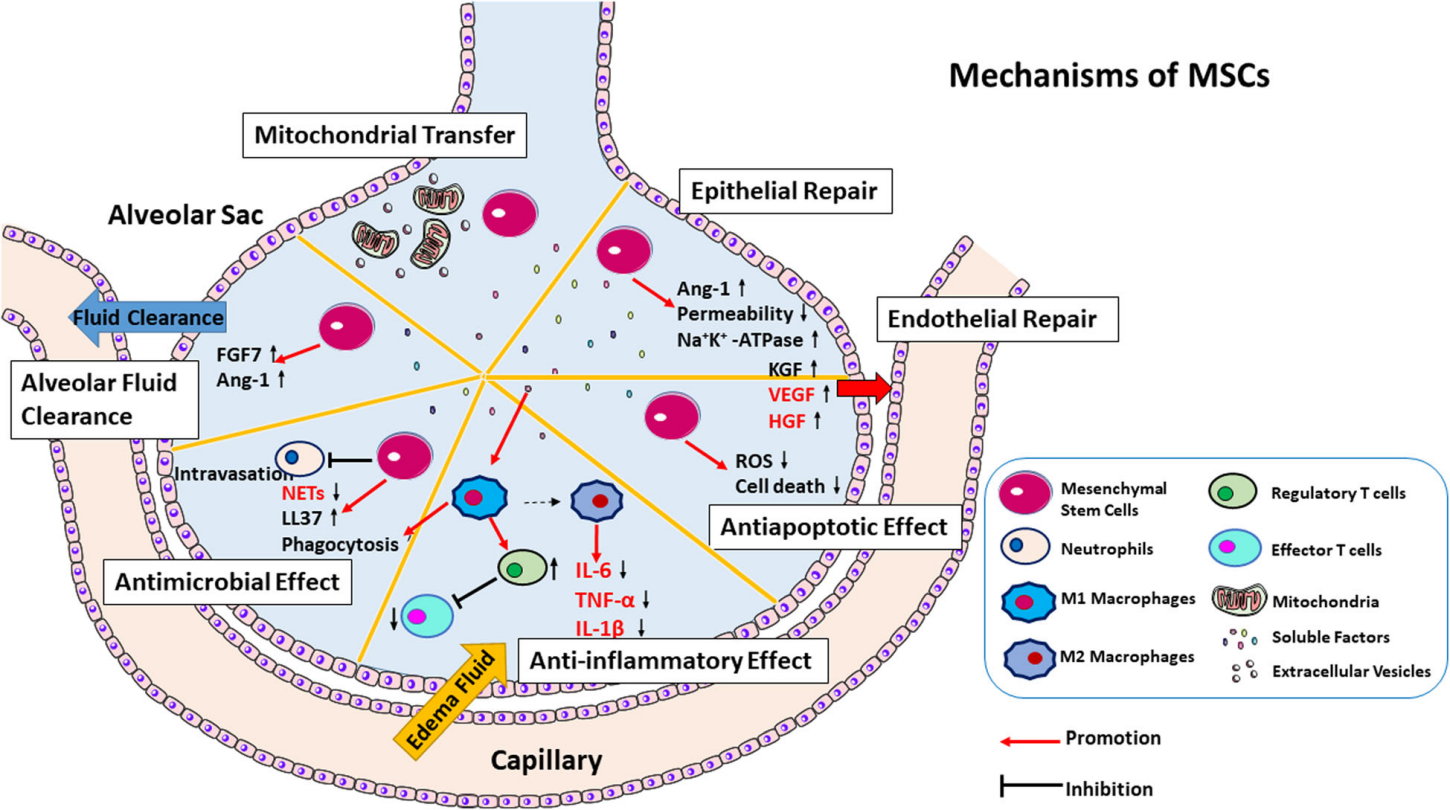
Schematic diagram of the mechanisms of action of MSCs in ARDS. MSCs act through the secretion of soluble factors and extracellular vesicles and the transfer of mitochondria. MSCs promote epithelial and endothelial repair, alveolar fluid clearance, and bacterial clearance and exert anti-inflammatory and antiapoptotic effects. MSCs release the peptide LL37 and inhibit neutrophil intravasation and NET formation, favoring bacterial clearance. In M1 macrophages, MSCs increase phagocytosis and promote bacterial clearance. MSCs activate regulatory T cells by inhibiting proliferation and activation. MSCs enhance the differentiation of macrophages to the M2 phenotype, produce anti-inflammatory cytokines, and inhibit the proinflammatory factors TNF-α, IL-6, and IL-1β, which is beneficial for tissue repair and may prevent the release of cytokine storms by the immune system. Simultaneously, Na+-K+-ATPase is upregulated in lung AT-II cells and inhibits fibrosis. By decoupling oxidative phosphorylation, MSCs reduce reactive oxygen species (ROS) levels and shift the metabolism to sugar metabolism, thereby promoting cell survival and reducing cell death. In addition, MSCs also promote the clearance of alveolar fluid by increasing the levels of fibroblast growth factor 7 (FGF7) and angiopoietin-1 (Ang-1). In addition to reducing the production of cytokine storms by the immune system, MSC therapy can also promote endogenous repair, restore the lung microenvironment of patients, protect alveolar epithelial cells, block pulmonary fibrosis, and treat COVID-19-associated pneumonia [26]. MSCs restored epithelial and endothelial permeability by releasing Ang-1. MSCs may promote the regeneration of type II alveolar epithelial cells by producing keratinocyte growth factor (KGF), vascular endothelial growth factor (VEGF), and hepatocyte growth factor (HGF); prevent the apoptosis of endothelial cells; and contribute to the repair of the alveolar epithelial barrier in ARDS-associated injury to enhance the repair of injured lung tissue in COVID-19 patients with ARDS. Text in a red font color indicates COVID-19-associated effects. EV, extracellular vesicles; NETs, neutrophil extracellular traps; Treg, regulatory T cells; AT-II, alveolar type II; ROS, reactive oxygen species; FGF7, fibroblast growth factor 7; Ang-1, angiopoietin-1

Although many efforts have been made to understand the therapeutic role of MSCs in ARDS, the mechanism of action has not yet been fully elucidated. Investigating the molecular mechanism of MSCs in the treatment of ARDS is of great importance for MSC-based cell therapy. Many preclinical studies have confirmed the therapeutic effect of MSCs in ARDS or ALI (Table S1). MSCs were initially thought to form niches for the propagation of hematopoietic stem cells that were extremely useful in confluent cultures. Initial research has also focused on the capability of MSCs to differentiate in vivo and transform into osteoblasts, chondrocytes, adipocytes, and even myoblasts. Therefore, MSCs can engraft at the site of tissue injury to participate in repairing the damage. Although refined research techniques have been applied, the effectiveness of MSC engraftment is still not satisfactory. The engraftment rates in lung injury models were low (< 1%) [31, 32]. Thus, studies have focused on the ability of MSCs to secrete paracrine factors such as immunoregulatory factors, angiogenic factors, antiapoptotic factors, and cell migration factors. These cytokines promote MSC migration and homing to damaged sites for repair. Other pathways have also been investigated, and it has been shown that MSCs interact with host tissue, including through mitochondrial transfer and direct interactions between cells. The beneficial effects of MSCs are mainly dependent on paracrine mechanisms [33]. Here, we described the potential therapeutic mechanisms of MSCs in ARDS (Fig. 2).

In patients with COVID-19, SARS-CoV-2 infects type II alveolar epithelial cells or other target cells that express ACE2. Chemokines secreted by these cells cause the influx of neutrophils, monocytes, and T cells. The accumulation of inflammatory cells in turn leads to the production of a large number of proinflammatory cytokines, known as a cytokine storm. Cytokine storms are the main cause of ARDS in COVID-19 patients [34]. Cytokine production was observed in many critically ill patients with COVID-19 [8, 35]. The key role of MSC treatment in COVID-19 is anti-inflammatory [36]. MSCs can reduce the production of proinflammatory cytokines, which may alleviate the cytokine storm induced by SARS-CoV-2, and increase the production of IL-10, which can reduce the inflow and aggregation of neutrophils in the lung and reduce the production of TNF-α [37]. KGF secreted by MSCs can reduce injury and promote proliferation and repair of alveolar epithelial cells by increasing surface-active substances, matrix metalloprotein (MMP)-9, IL-1Ra, GM-CSF, etc. [38]. According to the latest report, lung endothelial cells, as a therapeutic target in COVID-19, play important roles in the course of SARS-CoV-2 infection [39]. MSCs can release VEGF and HGF, which work together to stabilize endothelial barrier function by restoring pulmonary capillary permeability. By inhibiting pulmonary vascular endothelial cell apoptosis, enhancing the recovery of VE-cadherin, and reducing proinflammatory factors, MSCs control inflammation and protect the lung endothelial barrier [40]. Since SARS-CoV-2 mainly affects the lungs [41], the distribution of MSCs in the peripheral blood is mainly concentrated in the lungs after intravenous infusion [42], indicating that MSCs are a promising treatment option for patients with COVID-19 pneumonia.

## Clinical research progress

Currently, only a few reports have detailed the response of patients with acute respiratory distress syndrome to MSCs (Table S2). There have been some new findings in clinical trials completed in the last 5 years. Zheng et al.’s study (NCT01902082) was one of the earliest studies to detect the safety of mesenchymal stem cells in patients with ARDS. In this phase I, placebo-controlled study, no adverse reactions or serious adverse events were reported related to the infusion of MSCs between the two groups, and the length of hospital stay, days without the ventilator, and days without ICU care on the 28th day after treatment were similar. The serum SP-D level in the MSC group on the 5th day was significantly lower than that on day 0 (*p* = 0.027), and the IL-8 level was not significantly changed. The IL-6 level on day 5 showed a downward trend compared with that of day 0, but it was not statistically significant (*p* = 0.06). Therefore, the group concluded that allogeneic adipose-derived mesenchymal stem cells are safe for ARDS treatment. However, the clinical effects of using MSC doses are weak, and this strategy needs to be further optimized to achieve the satisfactory ARDS treatment [43].

In the RUMCESS trial (NCT01849237), the patients were randomly assigned (1:1) to receive either conventional therapy (CT) for septic shock (SS) or CT plus MSCs at a dose of 10^6^/kg intravenously within the first 10 h after SS onset. Except for one patient with myelodysplastic syndrome, all patients developed neutropenia after chemotherapy. Most of the positive blood cultures from patients were gram-negative. The APACHE II baseline scores were 34.2 and 32.2, and the sequential organ failure assessment (SOFA) scores were similar in both groups. After 3 months, 5 of the 8 patients in the CT + MSCs group who survived for 28 days died of sepsis-related organ dysfunction. As a result, MSC administration within the first few hours of SS may increase the short-term survival of patients with neutropenia but cannot prevent death from sepsis-related organ dysfunction over time [44].

The STem cells for ARDS Treatment (START) trial was a multicenter, open-label, dose-escalation, phase 1 clinical trial. In 2015, Wilson et al. published the results of their START trial (NCT01775774). In nine patients, there were no prespecified infusion-related events or treatment-related adverse events. Severe adverse events in three patients were subsequently noted several weeks after infusion but were thought to occur before MSC injection based on the MRI results. Nine patients with moderate-to-severe ARDS tolerated a single intravenous infusion of human BM-MSC derived from allogeneic bone marrow well. The authors believe that more patients are needed to thoroughly investigate the response of ARDS patients to MSCs [45].

In a follow-up study, Simonson et al. (NCT02097641) performed a detailed analysis of the immunomodulatory and proteomics characteristics of two patients with severe refractory ARDS. Two patients received 2 × 10^6^ cells per kilogram, and patients showed improvements in breathing, hemodynamics, and multiple organ failure. At the same time, a variety of lung and systemic inflammatory markers, including epithelial cell apoptosis, leakage of alveolar-capillary fluid, proinflammatory cytokines, microRNAs, and chemokines, also declined. The results of this study also suggest that the use of adoptively transplanted mesenchymal stem cells for lung protection in patients with ARDS is beneficial but has not been validated. Further research is needed [46].

Matthay et al. conducted a prospective, double-blind, multicenter, randomized phase 2a trial (NCT02097641) to assess the safety of MSCs in patients with moderate-to-severe ARDS. This group recruited ventilation patients with moderate-to-severe ARDS (partial oxygen pressure to fractional inhaled oxygen ratio < 27 kPa, positive end-expiratory pressure [PEEP] 8 cmH_2_O) at five university medical centers. There was no difference in the 28-day mortality between the MSC group and the placebo group. The results show that for patients with moderate-to-severe ARDS, a single intravenous dose of MSCs is safe [47]. Based on this result, Zhang and colleagues further studied the rationale for why the MSC-administered group exhibited an increased 28-day mortality (not significant). Their research demonstrated that MSC treatment might be beneficial or detrimental depending on the patient’s specific pulmonary microenvironment, which included the levels of IL-6 and fibronectin, and total antioxidant capacity (TAC). MSC treatment is protective in a mouse model with reduced concentrations of IL-6 and fibronectin and increased levels of TAC, while MSCs worsened injury in the opposite conditions. This result provided an important rationale for a precision medicine approach for MSC treatment [48].

## Conclusion

ARDS is a severe acute respiratory failure syndrome with a high mortality rate. Despite extensive research, there is currently no specific support for the treatment of ARDS. Because MSCs have obvious therapeutic effects, such as promoting the repair of epithelial and endothelial tissues, clearance of alveolar fluid and microorganisms, and anti-inflammatory and antiapoptotic effects, MSC-based cell therapy is a promising strategy for the treatment of ARDS. The safety and possible efficacy have been demonstrated in some ARDS patients. Limited clinical data have shown that systemic administration of MSCs can significantly alleviate lung injury in COVID-19 patients. With the development of research involving MSCs for the treatment of COVID-19, MSC therapy may be a strategy for responding to the current epidemic. The mechanism of action of MSCs is complex. The key mechanisms include paracrine mechanisms, extracellular vesicles, or direct contact of cells with metastatic cell contents. Although some progress has been made, there is not enough clinical evidence to prove the effectiveness of MSCs in the treatment of ARDS. The survival rate of the cells, homing efficiency, gene mutation after transplantation, and tumorigenicity are the current challenges regarding clinical applications. Therefore, large-scale, long-term, multicenter trials are needed to further explore the therapeutic effects and safety of MSCs.

## Supplementary information

**Additional file 1 **: **Table S1.** Summary of the therapeutic benefits of MSCs in current preclinical models of experimental ARDS/ALI. **Table S2.** Ongoing and new studies of mesenchymal stem cell-based therapies for COVID-19 pneumonia.

## Data Availability

All clinical trial data were accessed from the Project Data Sphere platform ClinicalTrials.gov (https://clinicaltrials.gov/).

## Abbreviations

ARDS: Acute respiratory distress syndrome
MSCs: Mesenchymal stromal cells
COVID-19: Coronavirus disease 2019
SARS-CoV-2: Severe acute respiratory syndrome coronavirus 2
ACE2: Angiotensin-converting enzyme 2
ALI: Acute lung injury
CT: Conventional therapy
SS: Septic shock
START: STem cells for ARDS Treatment
TAC: Total antioxidant capacity
Mar: Macrophage
TMPRSS2: Transmembrane protease serine 2
NK: Natural killer
IFN: Interferon
IL: Interleukin
GM-CSF: Granulocyte-macrophage colony-stimulating factor
TNF: Tumor necrosis factor
EV: Extracellular vesicles
NETs: Neutrophil extracellular traps
Treg: Regulatory T cell
AT-II: Alveolar type II
ROS: Reactive oxygen species
FGF7: Fibroblast growth factor 7
Ang-1: Angiopoietin-1
KGF: Keratinocyte growth factor
VEGF: Vascular endothelial growth factor
HGF: Hepatocyte growth factor
MMP: Matrix metalloprotein
SOFA: Sequential organ failure assessment
