# Supplementary material for: Mesenchymal stem cells: current clinical progress in ARDS and COVID-19
Source: Stem Cell Res Ther. 2020 Jul 22;11:305. doi: 10.1186/s13287-020-01804-6 (PMC7373844; doi:10.1186/s13287-020-01804-6)
Supplement: Supplementary file 1 — Additional file 1 : Table S1. Summary of the therapeutic benefits of MSCs in current preclinical models of experimental ARDS/ALI. Table S2. Ongoing and new studies of mesenchymal stem cell-based therapies for COVID-19 pneumonia. [file 13287_2020_1804_MOESM1_ESM.docx]

Table S1. Summary of therapeutic benefits of MSCs in current preclinical models of experimental ARDS/ALI.

| Article Source | Injury Model | MSC Source | MSC Delivery Method | Outcomes | Key Factors | References |
| --- | --- | --- | --- | --- | --- | --- |
| *Am J Resp Cell Mol* | Mice bleomycin | MBMDMSC | Intravenous 6h after injury | Proinflammatory cytokines↓ | IFN-γ,IL-2,IL-1,IL-4 | **[1]** |
| *J Immunol* | Mice E.coli endotoxin | MBMDMSC | Intratracheal 4h after injury | Survival↑  BAL markers of inflammation↓ | TNF-ɑ,MIP-2 | **[2]** |
| *PLoS Med* | Mice LPS | MBMDMSC | Intravenous 30min after injury | BAL markers of inflammation↓ | Angiopoietin 1 | **[3]** |
| *Proc Natl Acad Sci USA* | Mice bleomycin | MBMDMSC | Intravenous after injury | BAL neutrophils and TNF↓ | IL-1ra | **[4]** |
| *Nat Med* | Mice CLP | MBMDMSC | Intravenous 24h before, during, or 1h after injury | Survival↑  Plasma proinflammatory cytokines↓ | PGE2,IL-10 | **[5]** |
| *Proc Nat Acad Sci USA* | Human endotoxin | HBMDMSC | Intratracheal 1h after injury | Lung water↓ | FGF7 | **[6]** |
| *Am J Respir Crit Care Med* | Mice CLP | MBMDMSC | Intravenous 6h after injury | Survival↑  Plasma proinflammatory cytokines↓ | IL-6,IL-1β,IL-10, KC,JE,CCL5 | **[7]** |
| *Blood* | Mice peritonitis | HBMDMSC | Intraperitoneal 15min after injury | Intraperitoneal inflammatory cell infiltrate↓ | TSG-6 | **[8]** |
| *Stem Cell Res Ther* | Mice OA LPS | HBMDMSC | OA 4h after injury | BAL inflammatory cells and proinflammatory cytokines↓ | TSG-6 | **[9]** |
| *Thorax* | Rat VILI | RBMDMSC | Intravenous immediately and 24h after injury | BAL inflammatory cells and proinflammatory cytokines↓ | TNF-ɑ,IL-10 | **[10]** |
| *Am J Respir Crit Care Med* | Human E.coli | HBMDMSC | Intravenous or intrabroncheal 1h after injury | Neutrophil influx↓ | TNF-ɑ,IL-10 | **[11]** |
| *Crit Care Med* | Mice LPS | BMDMSC | Intravenous 24h after injury | MMP-8↑  TIMP-1 and lung inflammation↓ | IL-1β,IL-6,IL-10,IFN-γ,MCP-1,MIP-1α | **[12]** |
| *Thorax* | Sheep P.aeruginosa | HBMDMSC | Intratracheal 1h after injury | Oxygenation↑  Pulmonary edema↓ | PaO2/FiO2 ratio | **[13]** |
| *J Immunol* | Mice E.coli endotoxin | HBMDMSC | Intratracheal 4h after injury | Survival↑  LXA4↑  BAL proinflammatory cytokines↓ | LXA4 | **[14]** |
| *Thorax* | Rat E.coli | HBMDMSC | Intravenous 0.5h after injury | Survival, IL-10 and KGF levels↑  IL-6↓ | IL-10,KGF,LL-37 | **[15]** |
| *Proc Natl Acad Sci USA* | Mice influenza H5N1 | HBMDMSCs | Intravenous 4 post infection | Survival, Ang-1 and KGF levels↑  Lung inflammation, pulmonary edema and permeability↓ | IL-1β,TNF-α,IFN-γ | **[16]** |
| *Crit Care Med* | Mice LPS | BMDMSC | Intravenous 12h after injury | Survival↑  Pulmonary vascular permeability↓ | PGE_2_,IL-1β,TNF-a, IL-10 | **[17]** |
| *Am J Respir Crit Care Med* | Mice LPS | HBMDMSC | Intravenous 4h after injury | Pro-inflammatory cytokines↑  TNF-α, IL-8↓ | TNF-α,IL-8,IFN-γ, IL-1β,IL-17 | **[18]** |
| *Crit Care Med* | Rat LPS | BMDMSC | Intravenous 48h after injury | KGF levels↑  TNF-α, IL-1β,KC, and TGF-β levels↓ | KGF,IL-1β,TNF-α, TGF-β | **[19]** |
| *Crit Care* | Rabbit LPS | BMDMSC | Intravenous 24h after injury | O2 saturation, IL-10 level↑  Lung inflammation, pulmonary  edema, IL-6 and TNF-α levels↓ | IL-10,IL-6,TNF-α | **[20]** |
| *Am J Respir Crit Care Med* | Mice HCl instillation,  VILI, or both | BMDMSC | Intravenous 48h after injury | Fibrosis in HCl model↑  Fibrosis in VILI model alone↓ | IL-10,IL-6 | **[21]** |
| *J Clin Med* | Rat E.coli | UCDMSC | Intravenous 1h after injury | Survival↑  Pro-inflammatory cytokines↓  Alveolar tissue↓ | IL-10,IL-6,TNFα, MPC-1 | **[22]** |
| *Thorax* | Mice E.coli | MSC-MVs | Intravenous 1h after injury | Alveolar fluid clearance↑  Lung protein permeability↓ | TNFα | **[23]** |
| *Stem Cell Res Ther* | Rat E.coli | UC-hMSC | Intravenous 30 min after injury | lung tissue airspace↑  lung microvascular permeability, total infiltrating cell count↓ | IL-1β,IL-6 | **[24]** |
| *Stem Cell Res Ther* | Mice LPS | HCMSC | Tail vein inject 4 h after injury | Inflammatory cell infiltration, alveolar wall thickening, edema↓ | TNF-α,IL-1β,IL-6 | **[25]** |
| *Stem Cell Res Ther* | Rat bleomycin | MSC-MVs | Intratracheal 2d after injury | apoptosis and inflammation↓ | IL-6,IL-8,TNF-α | **[26]** |

**Abbreviation:** MSC mesenchymal stem (stromal) cell, AB antibody, Ang angiopoietin, BAL bronchoalveolar lavage, CLP caecal ligation and puncture, E.coli Escherichia coli, P.aeruginosa Pseudomonas aeruginosa, HBMDMSC human bone marrow-derived MSCs, HCMSC human chorionic villi-derived MSC, UC-hMSC umbilical cord-human MSCs, MBMDMSC murine bone marrow-derived MSCs, RBMDMSC rat bone marrow-derived MSCs, LPS lipopolysaccharide, MSC-CM MSC-conditioned media, OA oral aspiration, PGE2 prostaglandin E2, IL Interleukin, IL-1ra Interleukin 1 receptor antagonist, TNF tumour necrosis factor, VILI ventilator-induced lung injury, IFN Interferon, LXA4 lipoxin A4, KC murine IL8 homolog, JE murine MCP-1 homolog, CCL5 murine Rantes homolog, TIMP tissue inhibitor of metalloproteinase, MMP metalloproteinase, MVs Microvesicles, FGF7 fibroblast growth factor7, NA not applicable, h hour, min minute, d day.

**References**

1. Rojas M, Xu J, Woods CR, et al. Bone marrow-derived mesenchymal stem cells in repair of the injured lung. Am J Respir Cell Mol Biol. 2005;33:145–152, doi:10.1165/rcmb.2004-0330OC.

2. Gupta N, Su X, Popov B, et al. Intrapulmonary delivery of bone marrow-derived mesenchymal stem cells improves survival and attenuates endotoxin-induced acute lung injury in mice. J Immunol. 2007;179:1855–63, doi:10.4049/jimmunol.179.3.1855.

3. Mei SH, McCarter SD, Deng Y, et al. Prevention of LPS-induced acute lung injury in mice by mesenchymal stem cells overexpressing angiopoietin 1. PLoS Med. 2007;4:e269, doi:10.1371/journal.pmed.0040269.

4. Ortiz LA, Dutreil M, Fattman C, et al. Interleukin 1 receptor antagonist mediates the antiinflammatory and antifibrotic effect of mesenchymal stem cells during lung injury. Proc Natl Acad Sci USA. 2007;104:11002–07, doi:10.1073/pnas.0704421104.

5. Németh K, Leelahavanichkul A, Yuen PS, et al. Bone marrow stromal cells attenuate sepsis via prostaglandin E(2)-dependent reprogramming of host macrophages to increase their interleukin-10 production. Nat Med. 2009;15:42–49, doi:10.1038/nm.1905.

6. Lee JW, Fang X, Gupta N, et al. Allogeneic human mesenchymal stem cells for treatment of E. coli endotoxin-induced acute lung injury in the ex vivo perfused human lung. Proc Nat Acad Sci USA. 2009;106:16357–62, doi:10.1073/pnas.0907996106.

7. Mei SH, Haitsma JJ, Dos Santos CC, et al. Mesenchymal stem cells reduce inflammation while enhancing bacterial clearance and improving survival in sepsis. Am J Respir Crit Care Med. 2010;182:1047–57, doi:10.1164/rccm.201001-0010OC.

8. Choi H, Lee RH, Bazhanov N, et al. Anti-inflammatory protein TSG-6 secreted by activated MSCs attenuates zymosan-induced mouse peritonitis by decreasing TLR2/NF-κB signaling in resident macrophages. Blood. 2011;118:330–338, doi:10.1182/blood-2010-12-327353.

9. Danchuk S, Ylostalo JH, Hossain F, et al. Human multipotent stromal cells attenuate lipopolysaccharide-induced acute lung injury in mice via secretion of tumor necrosis factor-alpha-induced protein 6. Stem Cell Res Ther. 2011;2:27, doi:10.1186/scrt68.

10. Curley GF, Hayes M, Ansari B, et al. Mesenchymal stem cells enhance recovery and repair following ventilator-induced lung injury in the rat. Thorax. 2012;67:496–501, doi:10.1136/thoraxjnl-2011-201059.

11. Lee JW, Krasnodembskaya A, McKenna DH, et al. Therapeutic effects of human mesenchymal stem cells in ex vivo human lungs injured with live bacteria. Am J Respir Crit Care Med. 2013;187:751–60, doi:10.1164/rccm.201206-0990OC.

12. Maron-Gutierrez T, Silva JD, Asensi KD, et al. Effects of mesenchymal stem cell therapy on the time course of pulmonary remodeling depend on the etiology of lung injury in mice. Crit Care Med. 2013;41:e319–e333, doi:10.1097/CCM.0b013e31828a663e.

13. Asmussen S, Ito H, Traber DL, et al. Human mesenchymal stem cells reduced the severity of acute lung injury in a sheep model of bacterial pneumonia. Thorax. 2014;69:819–25, doi:10.1136/thoraxjnl-2013-204980.

14. Fang X, Abbott J, Cheng L, et al. Human Mesenchymal Stem (Stromal) Cells Promote the Resolution of Acute Lung Injury in Part through Lipoxin A4. J Immunol. 2015;195:875–881, doi:10.4049/jimmunol.1500244.

15. Devaney J, Horie S, Masterson C, et al. Human mesenchymal stromal cells decrease the severity of acute lung injury induced by E. coli in the rat. Thorax. 2015;70:625–35, doi:10.1136/thoraxjnl-2015-206813.

16. Chan MC, Kuok DI, Leung CY, et al. Human mesenchymal stromal cells reduce influenza A H5N1-associated acute lung injury in vitro and in vivo. Proc Nat Acad Sci USA. 2016;113:3621–3626, doi:10.1073/pnas.1601911113.

17. Han J, Lu X, Zou L, et al. E-Prostanoid 2 Receptor Overexpression Promotes Mesenchymal Stem Cell Attenuated Lung Injury. Hum Gene Ther. 2016;27:621–630, doi:10.1089/hum.2016.003.

18. Morrison TJ, Jackson MV, Cunningham EK, et al. Mesenchymal Stromal Cells Modulate Macrophages in Clinically Relevant Lung Injury Models by Extracellular Vesicle Mitochondrial Transfer. Am J Respir Crit Care Med. 2017;196:1275–1286, doi:10.1164/rccm.201701-0170OC.

19. Silva JD, Lopes-Pacheco M, Paz AHR, et al. Mesenchymal stem cells from bone marrow, adipose tissue, and lung tissue differentially mitigate lung and distal organ damage in experimental acute respiratory distress syndrome. Crit Care Med. 2018;46:e132–40, doi:10.1097/CCM.0000000000002833.

20. Mokhber Dezfouli MR, Jabbari Fakhr M, Sadeghian Chaleshtori S, et al. Intrapulmonary autologous transplant of bone marrow-derived mesenchymal stromal cells improves lipopolysaccharide-induced acute respiratory distress syndrome in rabbit. Crit Care. 2018;22:353, doi:10.1186/s13054-018-2272-x.

21. Islam D, Huang Y, Fanelli V, et al. Identification and Modulation of Microenvironment Is Crucial for Effective Mesenchymal Stromal Cell Therapy in Acute Lung Injury. Am J Respir Crit Care Med. 2019;199:1214–1224, doi:10.1164/rccm.201802-0356OC.

22. Jerkic M, Masterson C, Ormesher L, et al. Overexpression of IL-10 Enhances the Efficacy of Human Umbilical-Cord-Derived Mesenchymal Stromal Cells in E. coli Pneumosepsis. J Clin Med. 2019;8:847, doi:10.3390/jcm8060847.

23. Park J, Kim S, Lim H, et al. Therapeutic effects of human mesenchymal stem cell microvesicles in an ex vivo perfused human lung injured with severe E. coli pneumonia. Thorax. 2019;74:43–50, doi:10.1136/thoraxjnl-2018-211576.

24. Horie S, Masterson C, Brady J, et al. Umbilical cord-derived CD362+ mesenchymal stromal cells for E. coli pneumonia: impact of dose regimen, passage, cryopreservation, and antibiotic therapy. Stem Cell Res Ther. 2020;11:116, doi:10.1186/s13287-020-01624-8.

25. Feng Y, Wang L, Ma X, et al. Effect of hCMSCs and liraglutide combination in ALI through cAMP/PKAc/β-catenin signaling pathway. Stem Cell Res Ther. 2020;11:2, doi:10.1186/s13287-019-1492-6.

26. Chen WX, Zhou J, Zhou SS, et al. Microvesicles derived from human Wharton's jelly mesenchymal stem cells enhance autophagy and ameliorate acute lung injury via delivery of miR-100. Stem Cell Res Ther. 2020;11:113, doi:10.1186/s13287-020-01617-7.

Table 2. Ongoing and new studies of mesenchymal stem cell-based therapies in COVID-19 pneumonia.

| Registry Numbers | Country | | Phase | | Status | MSC Source | Delivery Method | Estimated Enrolment | Start Date | End Date | Main outcomes |
| --- | --- | --- | --- | --- | --- | --- | --- | --- | --- | --- | --- |
| ChiCTR2000029990 | China | | / | | Completed | BMDMSC | Intravenous 1x10^6^cells/kg | 10 | January 23,2020 | February 16,2020 | Improved the inflammation situation in severe COVID-19; improved outcomes. |
| Ongoing | |  | |  | |  |  |  |  |  |  |
| NCT04315987 | Brazil | | 1 | | Not yet recruiting | UC-MSCs | Intravenous 2x10^7^cells/kg | 66 | April  2020 | June  2020 | / |
| NCT04313322 | Jordan | | 1 | | Recruiting | WJ-MSCs | Intravenous 1x10e6/kg | 5 | March 16, 2020 | September 30, 2020 | / |
| NCT04333368 | France | | 1/2 | | Recruiting | UC-MSCs | Intravenous 1 Million/kg | 60 | April 6, 2020 | July 31,  2021 | / |
| NCT04339660 | China | | 1/2 | | Recruiting | UC-MSCs | Intravenous 1 x10E6 cells/kg | 30 | February 1,  2020 | June 30,  2020 | / |
| NCT04352803 | / | | 1 | | Not yet recruiting | AA-MSCs | Intravenous 500,000/kg | 20 | April  2020 | April  2026 | / |
| NCT04371601 | China | | 1 | | Active, not recruiting | UC-MSCs | Intravenous 10^6^/kg | 60 | March 1, 2020 | December 31, 2022 | / |
| NCT04361942 | Spain | | 2 | | Recruiting | Allogenic-MSCs | Intravenous 1 million cells/kg | 24 | April  2020 | December 31, 2020 | / |
| NCT04252118 | China | | 1 | | Recruiting | MSCs | Intravenous 3.0 x 10E7 cells/kg | 20 | January 27,2020 | December, 2021 | / |
| NCT04273646 | China | | / | | Not yet recruiting | UC-MSCs | Intravenous 5.0 x 10E6 cells/kg | 48 | April 20, 2020 | February 15,2022 | / |
| NCT04269525 | China | | 2 | | Recruiting | UC-MSCs | Intravenous 3.3 x 10^7^ cells/50ml | 10 | February 6,  2020 | September 30, 2020 | / |

**Abbreviation:** WJ-MSCs Wharton's Jelly-Mesenchymal Stem Cells, UC-MSCs umbilical cord-derived MSCs, AA MSCs Autologous Adipose Derived Mesenchymal Cells.
